# CSRP1 gene: a potential novel prognostic marker in acute myeloid leukemia with implications for immune response

**DOI:** 10.1007/s12672-024-01088-9

**Published:** 2024-06-27

**Authors:** Chunxia Zhao, Yulu Wang, Huan Wang, Amit Sharma, Yun Wu, Ingo G. H. Schmidt-Wolf, Zifeng Wang

**Affiliations:** 1 Department of Nursing, The Second Affiliated Hospital, Jiangxi Medical College, Nanchang University, Nanchang, China; 2 Jiangxi Provincial Key Laboratory of Hematological Diseases, Department of Hematology, The First Affiliated Hospital, Jiangxi Medical College, Nanchang University, Nanchang, China; 3 Department of Integrated Oncology, Center for Integrated Oncology (CIO), University Hospital of Bonn, Bonn, Germany; 4 Department of Neurosurgery, University Hospital of Bonn, Bonn, Germany; 5 Department of Hematology, Shangrao People’s Hospital, The Affiliated Shangrao Hospital of Nanchang University, Shangrao, China

**Keywords:** Acute myeloid leukemia, CSRP1, Immune, DNA-methyltransferase, Prognosis

## Abstract

**Background:**

Acute myeloid leukemia, constituting a majority of leukemias, grapples with a 24% 5-year survival rate. Recent strides in research have unveiled fresh targets for drug therapies. LIM-only, a pivotal transcription factor within LIM proteins, oversees cell development and is implicated in tumor formation. Among these critical LIM proteins, CSRP1, a Cysteine-rich protein, emerges as a significant player in various diseases. Despite its recognition as a potential prognostic factor and therapeutic target in various cancers, the specific link between CSRP1 and acute myeloid leukemia remains unexplored. Our previous work, identifying CSRP1 in a prognostic model for AML patients, instigates a dedicated exploration into the nuanced role of CSRP1 in acute myeloid leukemia.

**Methods:**

R tool was conducted to analyze the public data. qPCR was applied to evaluate the expression of CSRP1 mRNA for clinical samples and cell line. Unpaired t test, Wilcoxon Rank Sum test, KM curves, spearman correlation test and Pearson correlation test were included in this study.

**Results:**

CSRP1 displays notable expression variations between normal and tumor samples in acute myeloid leukemia (AML). It stands out as an independent prognostic factor for AML patients, showing correlations with clinical factors like age and cytogenetics risk. Additionally, CSRP1 correlates with immune-related pathways, immune cells, and immune checkpoints in AML. Furthermore, the alteration of CSRP1 mRNA levels is observed upon treatment with a DNMT1 inhibitor for THP1 cells.

**Conclusion:**

The CSRP1 has potential as a novel prognostic factor and appears to influence the immune response in acute myeloid leukemia. Additionally, there is an observed association between CSRP1 and DNA methylation in acute myeloid leukemia.

**Supplementary Information:**

The online version contains supplementary material available at 10.1007/s12672-024-01088-9.

## Background

Acute myeloid leukemia (AML), as one of the most common leukemias in adults, accounting for approximately 70% of all leukemias, has a 5-year survival rate of only 24% [1]. Current clinical approaches for AML treatment include traditional regimens such as "7 + 3" induction chemotherapy (cytarabine + daunorubicin), high-dose cytarabine consolidation, allogeneic hematopoietic stem cell transplantation, especially for elderly AML patients who often have characteristics like advanced age, multiple comorbidities, and high rates of adverse prognostic gene mutations, low-intensity chemotherapy and demethylation treatment are preferred [2]. With advances in basic and translational medical research, especially the use of large-scale genomic analysis to understand the molecular landscape of AML, the unique immunophenotype and gene mutations of leukemia cells provide precise targets for targeted drug therapies, such as Bcl-2, FLT3-ITD, and IDH1/2 inhibitors. These therapies offer hope for AML patients who cannot tolerate intensive chemotherapy or experience relapse or are difficult to treat [3]. Increasing research continues to reveal the underlying mechanisms and treatments for AML, such as the use of Hedgehog signaling pathway inhibitor glasdegib in the diagnosis and treatment of AML [4].

The LIM domain is a structure rich in cysteine and histidine, typically involved in protein binding and participating in biological processes such as cell signal transduction and gene transcription regulation [5]. LIM-only, as one of the main transcription factors of LIM proteins, regulates cell development, differentiation, and is involved in tumor formation [6]. CRPs (Cysteine-rich proteins) are a crucial family of LIM proteins, and CSRP1 protein, a member of the CRPs family, plays a significant role in various physiological and pathological processes. The increasing numbers of articles proved the CSRP1 is involved in predicting the survival of diverse cancers [7–12]. Besides, CSRP1 might be a target for Celecoxib against gastric cancer [13]. Thus, CSRP1 has the potential role as prognostic factor and therapy target.

Recently, the interaction of lncRNA NUDT6 with CSRP1 regulating cell motility and SMC (smooth muscle cell) differentiation in vascular disease was reported [14]. This evidence broadens the potential role of CSRP1 correlating with epigenetic factors. Additionally, a report indicates that CSRP1 is related to methylation in liver cancer [15], revealing that the correlation between CSRP1 and DNA methylation may affect tumor growth. DNA methylation is a crucial mechanism in epigenetics, mediated by DNA methyltransferases (DNMT1, DNMT3A, and DNMT3B), with DNMT1 playing a crucial role during DNA replication [16, 17]. Studies indicate that epigenetics plays a significant role in the pathogenesis of AML [18–20], with DNMT1 mediating gene suppression by increasing promoter methylation [21]. Additionally, DNMT1 has been shown to accelerate the progression and severity of AML by mediating the high methylation of tumor suppressor genes [22]. Thus, it is worth to investigate the interaction of CSRP1 with epigenetics, especially DNA methylation in AML.

Given the potential role of CSRP1 in various cancer types and our previous work [23], which identified a prognostic model containing DAXX, PSMB8, CSRP1, RAC2, and PTPN6, this current study is dedicated to investigating the role of the CSRP1 gene in acute myeloid leukemia (AML).

## Materials and method

### Gene expression, survival information and clinical indicators

In our current study, two AML datasets (TCGA-LAML and GSE65409) were included. Gene expression data of AML patients were downloaded from GEO database (https://www.ncbi.nlm.nih.gov/geo, dataset: GSE65409) and TCGA database (https://portal.gdc.cancer.gov/repository, project: TCGA-LAML). GSE65409 dataset has 8 health samples and 30 AML samples, while TCGA-LAML dataset has 150 AML samples. In addition, the clinical information of TCGA AML patients were collected from UCSC Xena database (https://xenabrowser.net/datapages/, corhort: GDC TCGA Acute Myeloid Leukemia (LAML)). For TCGA AML patients, 150 patients contain gene expression data, while among them 131 patients have survival information (survival time and survival statistics). After overlap with the patients who have clinical indicators, total 127 patients present both gene expression, survival information and clinical indicators (including age, gender, blast cells, bone marrow blast cells, leukocyte, hemoglobin and cytogenetics risk).

## Pan-cancer view of CSRP1 gene expression and investigation of its expression in AML samples of GEO data

The expression of CSRP1 gene were visualized by utilizing TIMER 2.0 (http://timer.cistrome.org/) in total 33 kinds of tumors including ACC (Adrenocortical Cancer), BLCA (Bladder Urothelial Carcinoma), BRCA (Breast invasive carcinoma), CESC (Cervical squamous cell carcinoma and endocervical adenocarcinoma), CHOL (Cholangiocarcinoma), COAD (Colon adenocarcinoma), DLBC (Lymphoid Neoplasm Diffuse Large B-cell Lymphoma), ESCA (Esophageal carcinoma), GBM (Glioblastoma multiforme), HNSC (Head and Neck squamous cell carcinoma), KICH (Kidney Chromophobe), KIRC (Kidney renal clear cell carcinoma), KIRP (Kidney renal papillary cell carcinoma), LAML (Acute Myeloid Leukemia), LGG (Brain Lower Grade Glioma), LIHC (Liver hepatocellular carcinoma), LUAD (Lung adenocarcinoma), LUSC (Lung squamous cell carcinoma), MESO (Mesothelioma), OV (Ovarian serous cystadenocarcinoma), PAAD (Pancreatic adenocarcinoma), PCPG (Pheochromocytoma and Paraganglioma), PRAD (Prostate adenocarcinoma), READ (Rectum adenocarcinoma), SARC (Sarcoma), SKCM (Skin Cutaneous Melanoma), STAD (Stomach adenocarcinoma), TGCT (Testicular Germ Cell Tumors), THCA (Thyroid carcinoma), THYM (Thymoma), UCEC (Uterine Corpus Endometrial Carcinoma), UCS (Uterine Carcinosarcoma) and UVM (Uveal Melanoma).

### Exploration of the prognostic ability of CSRP1 gene and association with clinical indicators in AML patients

The Kaplan–Meier method was applied to investigate the relation between CSRP1 gene expression and survival. ROC curves were performed to further confirm the robust prognosis of CSRP1 gene expression. Univariable and multivariable cox analysis were utilized to explore the independent prognostic risk factors. Wilcoxon Rank Sum test was applied to present the association of CSRP1 gene and clinical indicators. Besides 60 years old as the cutoff value of age, the respective median values of each other continuous clinical indicators (blast cells, bone marrow blast cells, leukocyte, hemoglobin) were set as their cutoff values to classify them into high and low groups. Thus, subgroups from clinical indicators including age (< 60 and ≥ 60), gender (male and female), blast cells (high and low), bone marrow blast cells (high and low), leukocyte (high and low), hemoglobin (high and low), and cytogenetics risk (favorable, normal and poor).

### Functional enrichment and immune relevant alteration in AML patients

R package clusterProfile was used to perform KEGG and GO enrichment analysis. ESTIMATE algorithm was applied to calculate the estimate score (representing tumor purity), immune score (representing the degree of infiltration of immune cells) and stromal score (representing the degree of stromal cells) for each sample. Then Wilcoxon Rank Sum test was used to compare these three scores between high and low CSRP1 gene expression groups. CIBERSORT method was applied to evaluate the relative percentage of 22 different types immune cells in each samples. Then, Wilcoxon Rank Sum test was used to compare the fractions of each immune cells between high and low CSRP1 gene expression groups. Spearman method was utilized to explore the correlation of CSRP1 gene expression and the fraction of each immune cells. The correlation of CSRP1 gene with immune checkpoint genes was evaluated by pearson analysis. Of note, the cutoff value for CSRP1 gene expression is the median gene expression value which is used to classify patients into high and low CSRP1 gene expression groups.

### The correlation of CSRP1 gene and DNMT family genes

To assess the correlation between the CSRP1 gene and DNMT family genes, we employed Pearson correlation analysis. Only correlations with p-value less than 0.05 were deemed significant.

### Clinical samples, RNA isolation and qPCR

For clinical samples, total 18 health samples and 18 AML samples were collected from Shangrao people’s hospital. The sample type for these clinical samples are bone marrow. Total RNA was extracted samples with Trizol (Takara Bio Inc, Tokyo, Japan) reagents according to the manufacturer's instructions. cDNA was prepared using the PrimeScript RT reagent kit with gDNA Eraser (Takara Bio Inc) as suggested by the protocol. Relative quantitation was performed using TB Green Premix Ex Taq (Takara Bio Inc). Quantitative PCR (qPCR) was performed on the Applied Biosystems 7500 Real Time PCR System (Applied Biosystems, Foster City, CA, USA). Relative quantitation was performed using TB Green Premix Ex Taq (Takara Bio Inc)with the following amplification program: 95 °C for 30 s, followed by 40 cycles of 95 °C for 5 s and 60 °C for 34 s. All the results were normalized to the level of GAPDH mRNA. The relevant primer sequences used were as follows:

- CSRP1-Fw, 5’-GAGGTTCAGTGCGAAGGCAACA-3’,
- CSRP1-Rev, 5’-CTTCTTGCCGTAGCAGGACTTG-3’,
- GAPDH-Fw, 5’-GACTCATGACCACAGTCCATGC-3’,
- GAPDH-Rev, 5’-AGAGGCAGGGATGATGTTCTG-3’.

This research was approved by the Ethics Committee of Shangrao people’s Hospital. Informed consent was acquired from all the individuals involved.

### Compound, cell culture, RNA isolation and qPCR

5-Azacytidine (AZA) (HY-10586) were purchased from MCE (MedChemExpress, NJ, USA) and dissolved in DMSO to10mM. The human AML cell line, THP1, was procured from the National Collection of Authenticated Cell Cultures in China. THP1 cells were cultured in RPMI 1640 medium supplemented with 10% fetal bovine serum (FBS), 2 uM L-glutamine, 100 U/mL penicillin, and 100 ug/mL streptomycin in a 5% CO2 atmosphere at 37 °C. Total RNA extraction and reverse transcription were carried out using the RNeasy Mini Kit (QIAGEN, Hilden, Germany) and SuperScript III First-Strand Synthesis kit (Invitrogen), respectively. Quantitative PCR (qPCR) was performed on the Applied Biosystems 7300 or 7500 Real Time PCR System (Applied Biosystems, Foster City, CA, USA). The PCR reaction utilized SYBR green PCR Master Mix (Applied Biosystems) with the following amplification program: 95 °C for 10 min, followed by 40 cycles of 95 °C for 15 s and 60 °C for 1 min. GAPDH mRNA served as the invariant control, and expression values were normalized to GAPDH. The specific primers used were as previously described.

### Statistical analysis

Graphpad prism was used to draw the figures and make statistical analysis for experiments. For bioinformatic part, R software was applied to visualize the figures and perform analysis. Unpaired t test, Wilcoxon Rank Sum test, KM curves, spearman correlation test and Pearson correlation test were included in this study. A p < 0.05 was considered as significant. *p < 0.05; **p < 0.01; ***p < 0.001; ns: not significant.

## Results

### Pan-cancer view for CSRP1 gene and its differential expression between healthy and AML samples

**Fig. 1:**
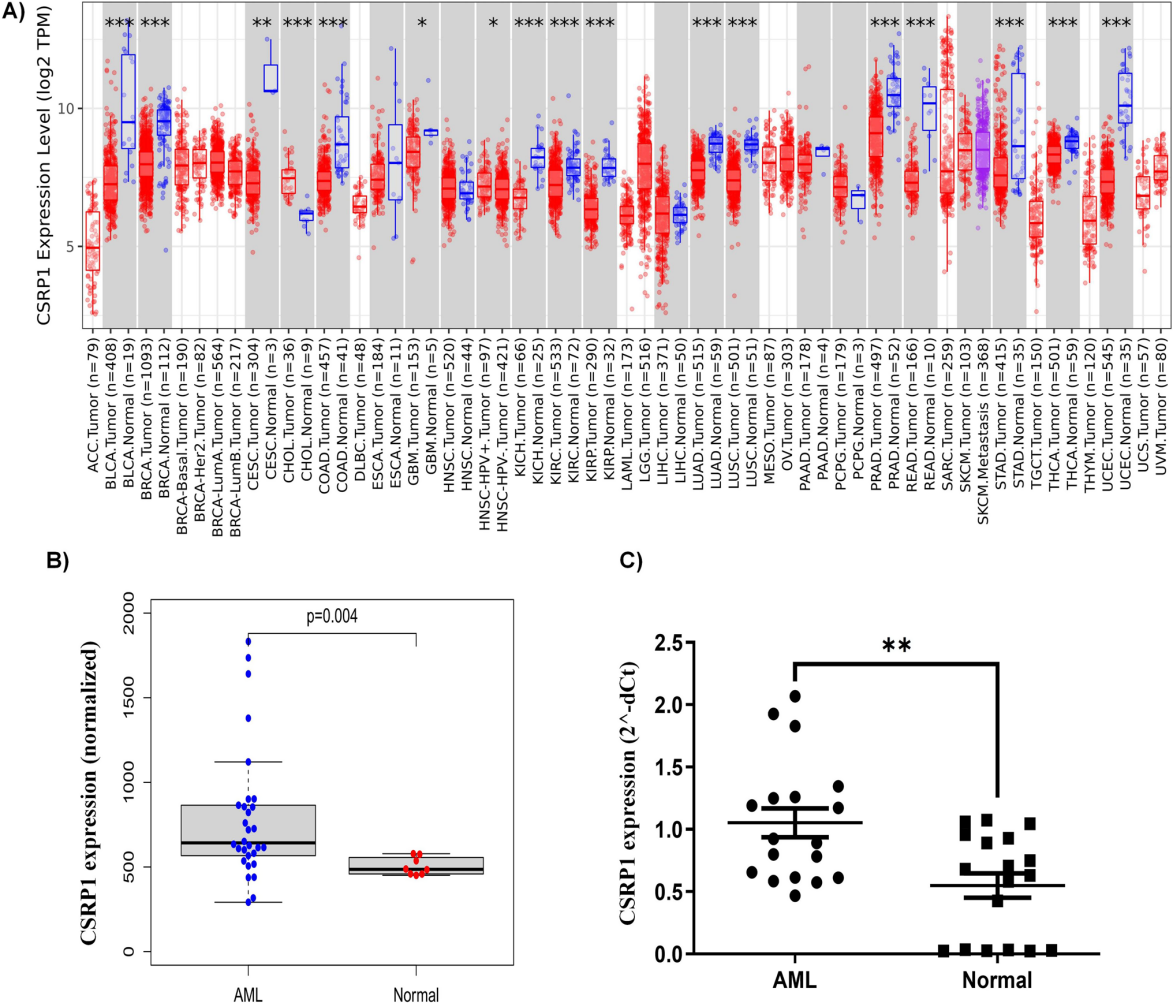
**A** The overview of CSRP1 gene expression in diverse cancers. Altered CSRP1 gene expression between AML and normal samples from GEO dataset (**B**) and our clinical samples (**C**)

We visualized the CSRP1 gene expression in 33 types of tumors (Fig. 1A), among them 21 kinds of tumors (BLCA, BRCA, CESC, CHOL, COAD, ESCA, GBM, HNSC, KICH, KIRC, KIRP, LICH, LUAD, LUSC, PAAD, PCPG, PRAD, READ, STAD, THCA and UCEC) have normal samples and tumor samples. Interestingly, 16 kinds of tumors (BLCA, BRCA, CESC, CHOL, COAD, GBM, KICH, KIRC, KIRP, LUAD, LUSC, PRAD, READ, STAD, THCA and UCEC) showed significantly differential CSRP1 gene expression between normal and tumor samples. Considering there is no comparison of normal and AML samples in this database, then we used the GEO data (GSE65409) and clinical samples to investigate the CSRP1 gene expression between normal and AML samples. In GEO data, the CSRP1 gene expression is significantly upregulated (p = 0.004) in AML samples compared to normal samples (Fig. 1B). Subsequently, we validated these findings in clinical samples, revealing that AML samples exhibited significantly higher CSRP1 gene expression (p = 0.002) compared to normal samples (Fig. 1C).

In both GEO data (GSE65409) and clinical dataset, we observed a small subset of samples exhibiting the highest expression levels. To delve deeper into this disparity, we conducted an analysis using GEO data (GSE65409). We segregated the samples with the highest expression levels from the entire dataset using a cut-off value of 1000 for the CSRP1 gene. The genes meeting the criteria (logFC ≥ 0.5 and P value < 0.05) were designated as differential genes. In the ensuing analysis, it becomes evident that certain genes display differential expression between the two groups, accompanied by associated pathways (supplementary Fig. 1). Nonetheless, the precise association of CSRP1 with these genes and pathways remains ambiguous. Further investigation is needed to unravel this connection and elucidate the underlying mechanism.

### CSRP1 gene as an independent prognostic factor and its relevant clinical indicators in AML

**Fig. 2:**
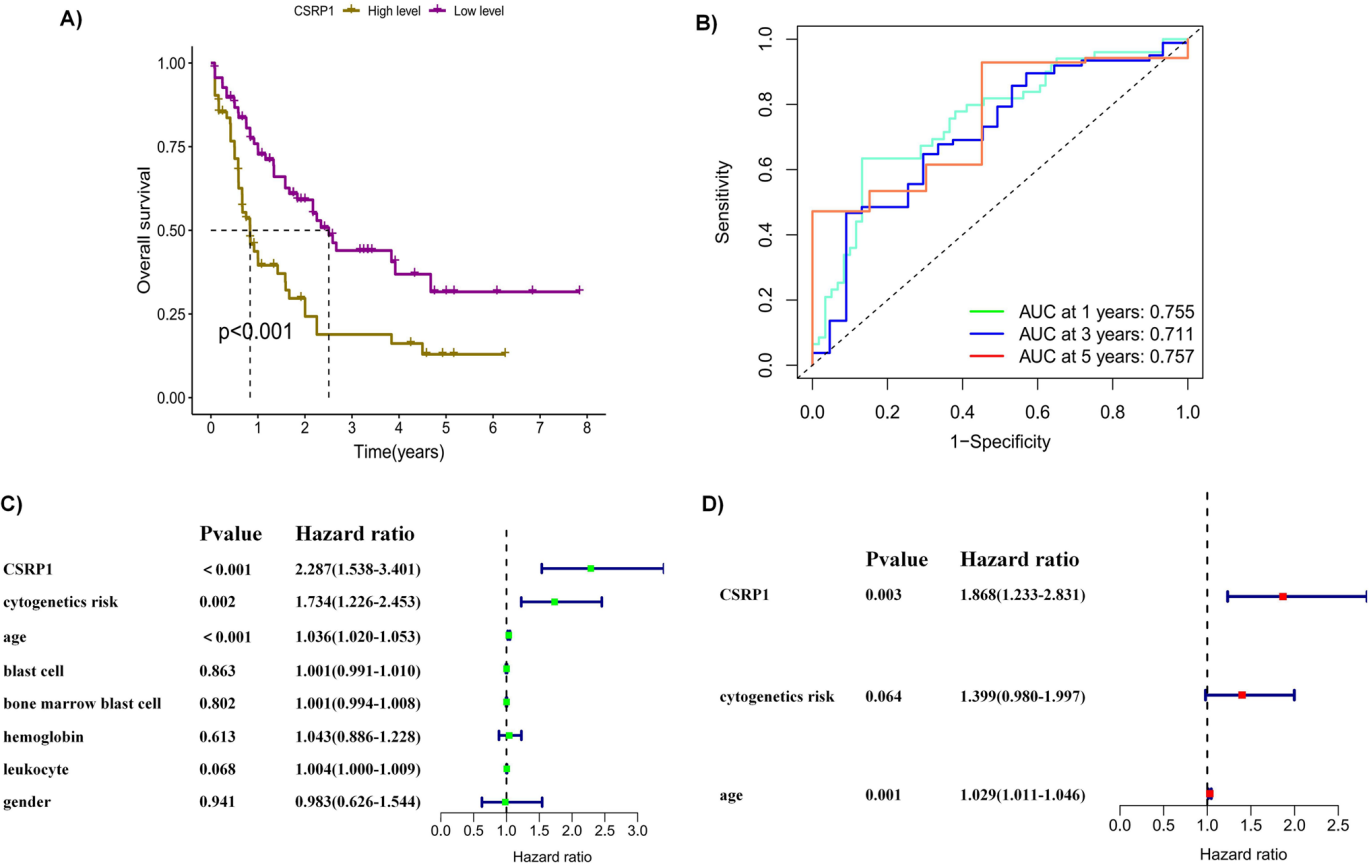
**A** Kaplan–Meier (KM) curve indicating that high CSRP1 gene expression is associated with low survival in AML patients. **B** Receiver Operating Characteristic (ROC) curve for CSRP1, predicting the survival of AML patients at different time points. **C** Univariate Cox regression analysis. **D** Multivariate Cox regression analysis demonstrating CSRP1 and age as independent prognostic factors

**Fig. 3:**
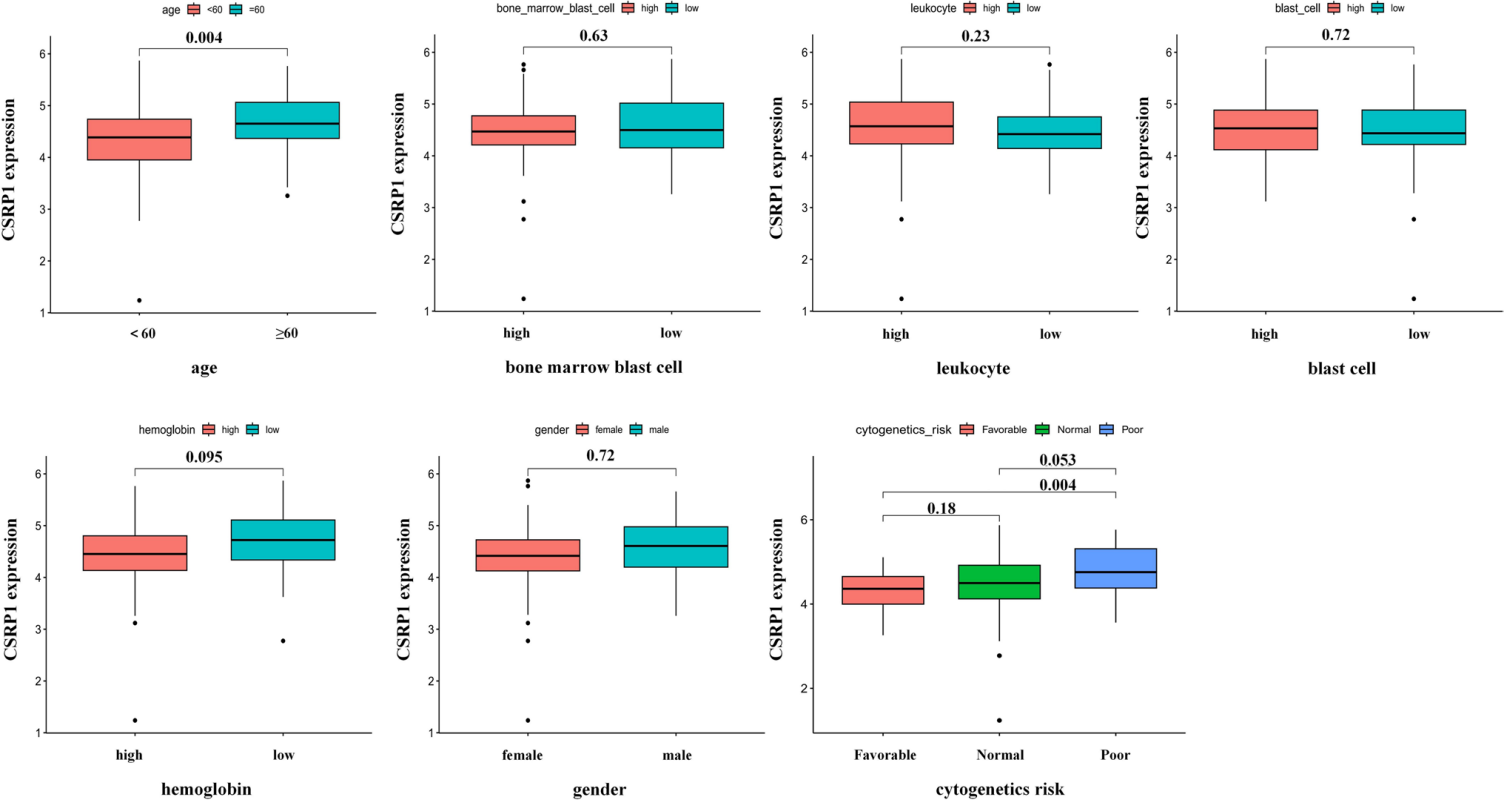
Comparison of CSRP1 gene expression between differential clinical sub-groups

By comparing the survival probability between high CSRP1 gene expression group and low CSRP1 gene expression group using KM curves, high group indicated a poor prognosis (p < 0.001) (Fig. 2A). In addition, ROC curves present that the AUC (area under the curve) values are 0.755 (1 year), 0.711 (3 years) and 0.757 (3 years) respectively at different predicting time points (Fig. 2B). All AUC values are both more than 0.7 confirming that CSRP1 is a robust prognostic factor for AML patients. Then, CSRP1 is combined with clinical indicators to determine the independent prognostic factor. Univariable cox analysis demonstrated CSRP1, age and cytogenetics risk are survival associated indicators, while only two independent factors, CSRP1 and age, were established by performing multivariable cox analysis (Fig. 2C, D). Overall, CSRP1 gene is an independent indicator for predicting the survival probability of AML patients. Besides, we explore the correlations of CSRP1 with clinical indicators (Fig. 3). For age, age no less than 60 subgroup showed higher CRSP1 gene expression compared to age less than 60 subgroup (p = 0.004). For cytogenetics risk, poor subgroup indicated high CSRP1 gene expression compared with favorable subgroup (p = 0.004). But there are no significant differential CSRP1 gene expression between the subgroups of other clinical indicators. Overall, CSRP1 gene expression correlated with some clinical indicators including age, and cytogenetics risk. Of note, the method to classify the subgroups in each clinical indicators has been mentioned in method part.

### CSRP1 relevant tumor microenvironment (TME), infiltrated immune cells, pathways and immune checkpoint genes

**Fig. 4:**
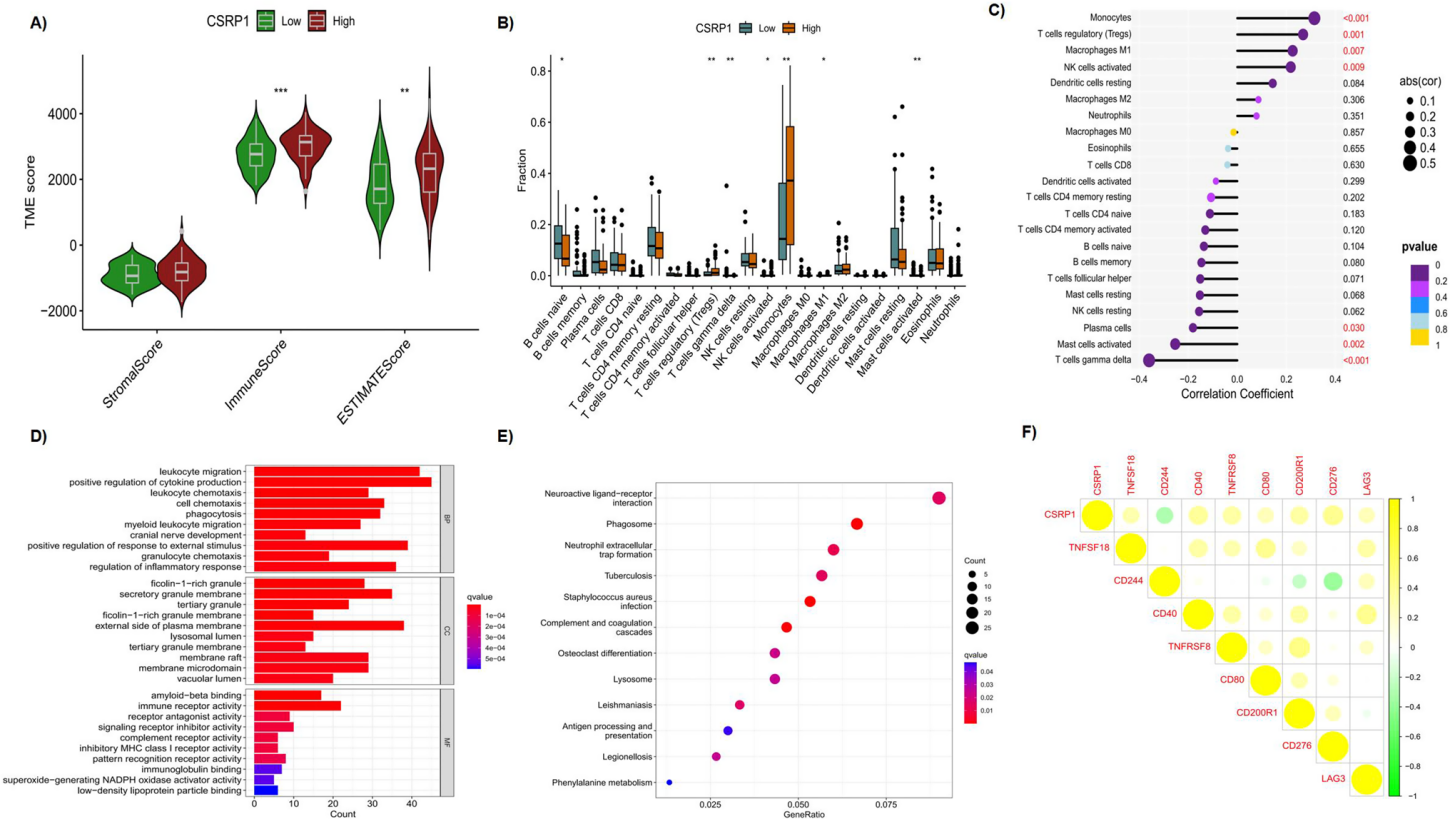
**A** Stromal, immune, and ESTIMATE scores related to CSRP1 using ESTIMATE analysis. **B** Comparison of the fraction of 22 different types of immune cells between high and low CSRP1 gene expression groups. **C** Investigating the association between CSRP1 gene expression and immune cells. **D** Gene Ontology (GO) enrichment analysis. **E** Kyoto Encyclopedia of Genes and Genomes (KEGG) pathway enrichment analysis. **F** Exploring the relationship between CSRP1 and checkpoint-related genes

Due to the important role of TME in cancers, we applied ESTIMATE algorithm to calculate the values of estimate score (tumor purity), immune score (infiltration of immune cells) and stromal score (the degree of stromal cells) for each sample. Then values of these three scores in CSRP1 subgroups (high and low CSRP1 gene expression subgroups) were accumulated. As shown in Fig. 4A, immune score and estimate score both present significant higher values in high CSRP1 subgroup compared to low CSRP1 subgroup (both p < 0.05), while stromal score showed no alteration between thes two CSRP1 subgroups. Considering immune score represent the infiltration of immune cells, we utilized CIBERSORT method to evaluate the fraction of 22 types of immune cells in each sample. By compared the fraction of 22 kinds immune cells between low and high CSRP1 subgroups, only 7/22 immune cells showed the significant differential percentages were observed (Fig. 4B). Among them, B cells naive, T cells gamma delta, Mast cells activated were decreased in high CSRP1 subgroup, while T cells regulatory, NK cells activated, Monocytes, Macrophages M2 were increased in high CSRP1 subgroup. The spearman correlation analysis was performed to further explore the correlation between CSRP1 gene and immune cells according to the criterion (|R|≥ 0.3, p < 0.05). Then T cells gamma delta (R =  − 0.36, p < 0.001) and Monocytes (R = 0.31, p < 0.001) were found correlated with CSRP1 gene (Fig. 4C, supplementary Fig. 2). In order to investigate the potential pathways involved in CSRP1 gene, GO and KEGG enrichment were performed. As shown in Fig. 4D and E, some immune related pathways/progress were collected which further support the immune relevant role of CSRP1 gene. Besides, the correlation between CSRP1 gene and 47 immune checkpoint genes were concluded by using Pearson correlation analysis according to the criterion (p < 0.001) (Fig. 4F). Interestingly, eight immune checkpoint genes (TNFSF18: R = 0.30, CD244: R = − 0.30, CD40: R = 0.38, TNFRSF8: R = 0.36, CD80: R = 0.27, CD200R1: R = 0.35, CD276: R = 0.43, LAG3: R = 0.27) were related with CSRP1 gene.

### DNMT1 correlated with CSRP1 gene expression

**Fig. 5:**
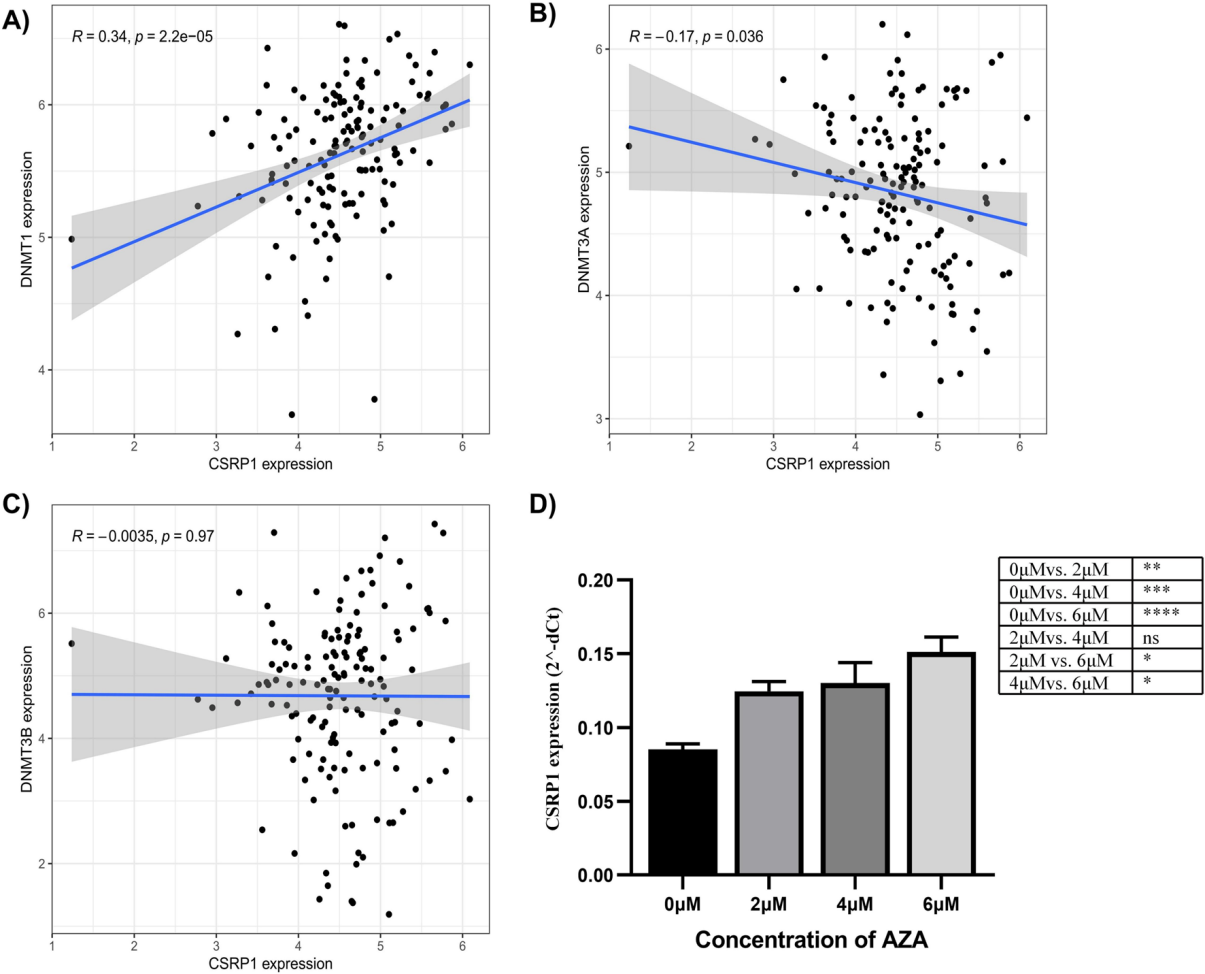
The correlation between CSRP1 and DNMT1 (**A**), DNMT3A (**B**), and DNMT3B (**C**) in AML patients. **D** THP1 cells treated with varying concentrations of the AZA compound (2uM, 4uM and 6uM), p value were calculated by two-way ANOVA and Tukey’s post-hoc test, the data represent the mean ± SD of triplicates per condition and are from one representative out of three independent experiments

In order to investigate the role of CSRP1 gene in AML, the correlation between DNMT family genes (DNMT1, DNMT3a and DNMT3b) with CSRP1 gene was performed. DNMT1 (r = 0.339, p < 0.001) and DNMT3A (r = − 0.171, p = 0.036) correlated with CSRP1 were observed as shown in Table 1 and Fig. 5A−C. To prove this concept, we added DNMT1 inhibitor (AZA compound) to THP1 cells and evaluated the CSRP1 gene expression level. We cocultured AML cell line THP1 with different concentrations (2uM, 4uM, 6uM and 8uM), and the mRNA level of CSRP1 was upregulated with the increasing amount of AZA compound (Fig. 5D and supplementary Fig. 3).

**Table 1:** The correlation between CSRP1 gene and DNMT family genes

Gene 1	Gene 2	r	p value
CSRP1	DNMT1	0.339	p<0.001
CSRP1	DNMT3A	− 0.171	0.036
CSRP1	DNMT3B	− 0.004	0.966

## Discussion

Recently, CSRP1 has been proven as a prognostic gene in many cancers including cholangiocarcinoma [10], prostate cancer [11, 24] and bladder cancer [25]. In addition, celecoxib present anti-cancer effect might contribute to inhibit CSRP1 gene expression in gastric cancer [13]. By screening the genes on 1q31.1–32.1 in Chinese patients, CSRP1 might be involved in the progression of sporadic colorectal cancer [26].

Considering CSRP1 has been identified as a potential biomarker in various cancers, we initially examined its expression across several cancer types. Among 20 types of tumors with both normal and tumor samples, alterations in CSRP1 expression were observed in 16 types when comparing normal and tumor samples. However, information on AML was limited due to the absence of normal samples. To address this, we investigated CSRP1 gene expression in both GEO data and clinical samples of AML. Interestingly, the results consistently indicated elevated CSRP1 gene expression in AML patients. This finding underscores its potential prognostic significance in AML, prompting further analyses to delve deeper into the role of CSRP1 in this context. Subsequent investigations revealed that AML patients with high CSRP1 gene expression exhibited lower survival probabilities, establishing it as an independent predictor for AML outcomes. Additionally, CSRP1 gene expression correlated with several clinical indicators, including age and cytogenetics risk, providing further support for the potential prognostic role of CSRP1 in AML.

As the tumor microenvironment (TME) plays a crucial role in AML, we employed the ESTIMATE algorithm to assess the relationship between CSRP1 and TME. The estimate score (indicating tumor purity) and immune score (reflecting the degree of immune cell infiltration), exhibited variations between CSRP1 high and low gene expression groups, suggesting distinct TME characteristics associated with CSRP1 gene. Notably, immune score differences indicated varying infiltrations of immune cells between CSRP1 high and low gene expression groups. Given that immune cell infiltration in tumors contributes to immune escape and resistance to immune therapy, we further examined the altered immune cells. In the high CSRP1 subgroup, B cells naive, T cells gamma delta, and Mast cells activated were decreased, while T cells regulatory, NK cells activated, Monocytes, and Macrophages M2 were increased compared to the low CSRP1 subgroup. It is noteworthy that a published paper reports the pivotal roles of Regulatory T cells and M2 macrophages in establishing the leukemic niche, thereby facilitating the shelter and proliferation of malignant clones [27]. In addition, spearman correlation analysis revealed a negative correlation with T cells gamma delta (R =  − 0.36, p < 0.001) and a positive correlation with Monocytes (R = 0.31, p < 0.001) concerning CSRP1 gene. Thus, CSRP1 is primarily associated with T cells gamma delta and monocytes, both proven to be involved in the survival and treatment outcomes of AML patients. γδ T cell-related therapies show promising potential for AML patients as immunotherapies [28, 29]. Several studies have demonstrated that absolute monocyte counts are negatively associated with AML patients’ survival [30–32]. Moreover, one study indicated that the combination of absolute monocyte count and absolute lymphocyte count can predict the survival of AML patients [33]. Subsequently, we applied GO and KEGG enrichment to evaluate potential pathways. Interestingly, only immune-relevant pathways were observed, while no direct tumor-related pathways were identified. This result suggests that the impact of the CSRP1 gene on AML patients may primarily occur through immune-related mechanisms. Currently, immune checkpoint inhibitors are promising therapies in the clinic. We explored the correlation of CSRP1 with immune checkpoint genes and observed associations with eight immune checkpoint genes (TNFSF18: R = 0.30, CD244: R =  − 0.30, CD40: R = 0.38, TNFRSF8: R = 0.36, CD80: R = 0.27, CD200R1: R = 0.35, CD276: R = 0.43, LAG3: R = 0.27). Described in a study, the CTLA4/CD80-86 Pathway, CD200/CD200R, LAG3, and CD276 have been identified or tested as promising targets in AML [34]. The alterations in these CSRP1-related genes may form a complex network affecting the immune response in AML patients at the genetic level, contributing to differential sensitivity to immunotherapies. Further investigation focusing on CSRP1 and its combination with immune checkpoint blocking treatments may provide benefits for AML patients. In summary, the correlation of CSRP1 with immune response in AML patients suggests its potential association with the tumor microenvironment (TME), likely impacting infiltrating immune cells and modulating immune checkpoint genes/proteins through diverse mechanisms.

Since DNMT1 is dysregulated in cancer, AML is no different to this scenario. Moreover, there are sufficient evidences that DNMT1 is a critical regulator in AML, yet its dynamics remain unclear [35, 36]. Intriguingly, it has been shown that treatment with 5-aza-2'-deoxycytidine (DNMT inhibitor) resulted in altered gene expression and methylation of the CSRP1 gene in HCC [15]. In the current study, we first investigated the relationship between genes of the DNMT family (DNMT1, DNMT2 and DNMT3) and CSRP1. The correlation analysis showed that the DNMT1 gene was highly associated with CSRP1 genes. When the DNMT1 inhibitor (AZA) was tested in the THP1 cell line, an increase in CSRP1 mRNA was observed, indicating a presumed alteration in the promoter methylation levels. While we have identified a possible link between DNMT1 and CSRP1, the exact mechanism is unclear. It will be of future interest to delineate to which extent DNMT1 modulates the activities of CSRP1.

Very recently, using independent sources and datasets, Hao et al. also observed high expression of CSRP1 in adult AML and have discussed its correlation with poor prognosis [37]. Thereby, further confirmed our findings. Of importance, the relationship between CSRP1 and the tumor microenvironment (TME) in AML patients described in our results provides additional insight and solidified the significance of CSRP1 as a prognostic indicator for AML patients.

## Conclusion

The CSRP1 gene emerges as a potential novel prognostic factor for individuals with acute myeloid leukemia (AML). Furthermore, it appears to influence the immune response in the context of AML. Additionally, there is an observed association between CSRP1 and DNA methylation. However, further exploration is needed to understand the mechanism of CSRP1 and its interaction with DNMT1 in AML cells.

### Supplementary Information

Additional file 1. (DOCX 231 KB)

## Data Availability

Public data were download from GEO database (https://www.ncbi.nlm.nih.gov/geo, dataset: GSE65409), TCGA database (https://portal.gdc.cancer.gov/repository, project: TCGA-LAML) and UCSC Xena database (https://xenabrowser.net/datapages/, corhort: GDC TCGA Acute Myeloid Leukemia (LAML)).
