# Supplementary material for: CSRP1 gene: a potential novel prognostic marker in acute myeloid leukemia with implications for immune response
Source: Discov Oncol. 2024 Jun 27;15:248. doi: 10.1007/s12672-024-01088-9 (PMC11211298; doi:10.1007/s12672-024-01088-9)
Supplement: Supplementary file 1 — Additional file 1. (DOCX 231 KB) [file 12672_2024_1088_MOESM1_ESM.docx]

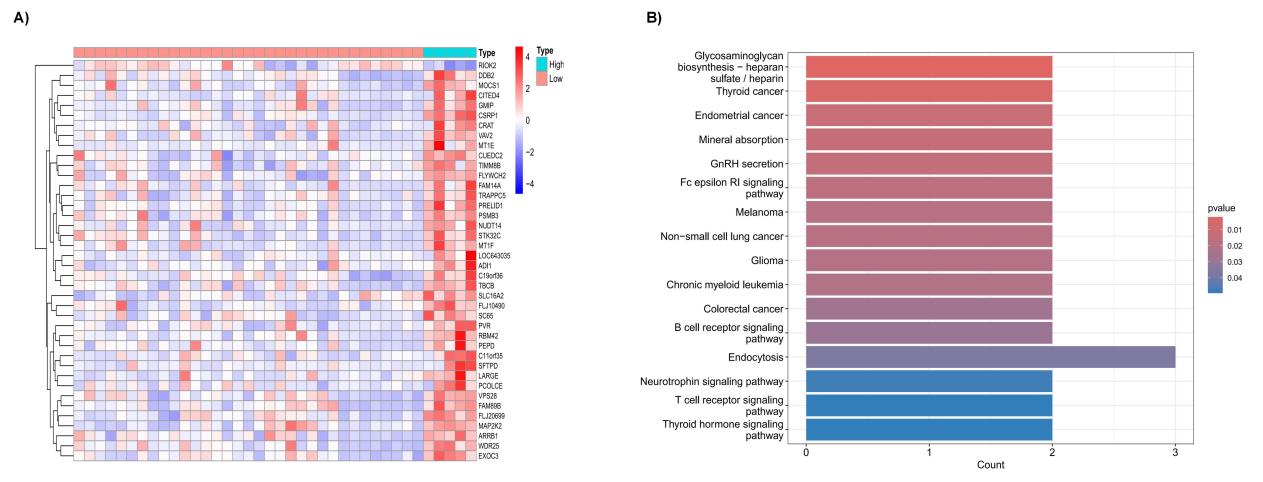


**Supplementary Figure 1.** Contrasting samples exhibiting high expression of CSRP1 (gene expression > 1000) with those showing lower expression (CSRP1 gene expression ≤ 1000): A) Differential gene expression profile. B) KEGG analysis.


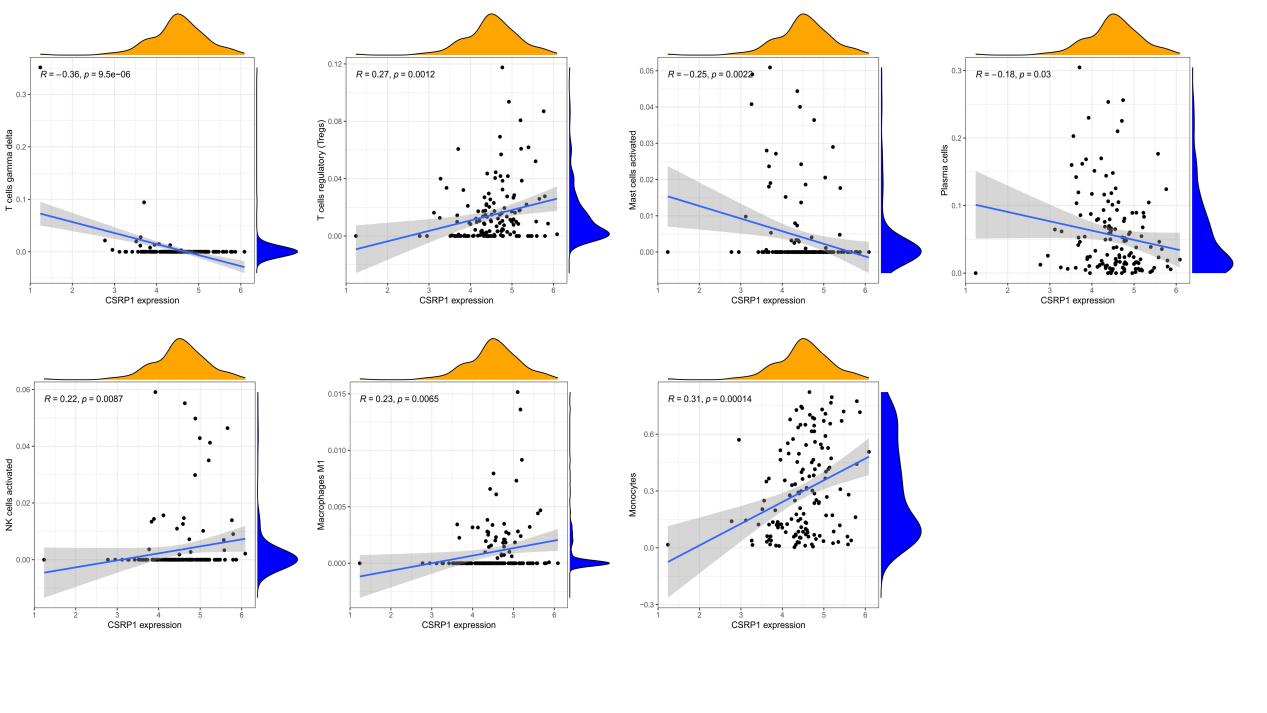


**Supplementary Figure 2.** The correlation of CSRP1 gene and diverse immune cells.


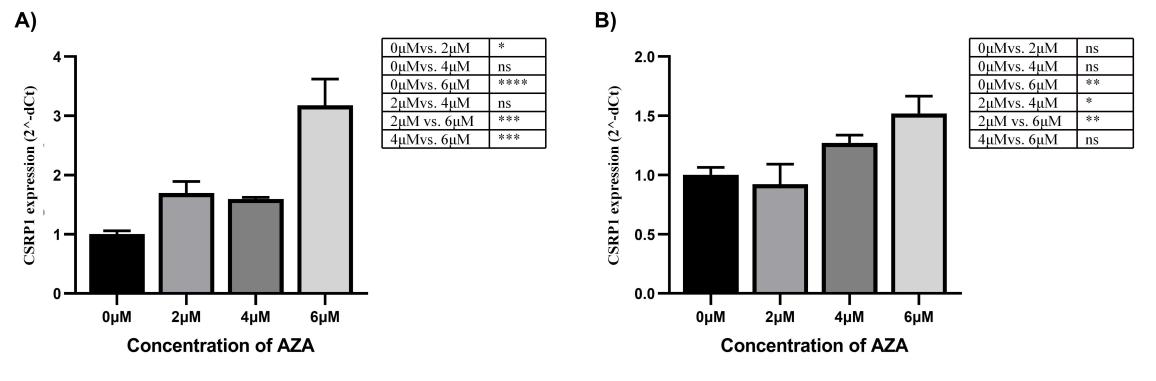


**Supplementary Figure 3.** THP1 cells treated with varying concentrations of the AZA compound (2uM, 4uM and 6uM), p value were calculated by two-way ANOVA and Tukey’s post-hoc test, the data represent the mean ± SD of triplicates per condition and are from two representative out of three independent experiments.
